# Differences in Thyroid Autoimmunity and Thyroid Function Tests Between Individuals with and without Obesity: Is There a Correlation with Obesity Degree?

**DOI:** 10.2174/0118715303342780250219111457

**Published:** 2025-03-20

**Authors:** Seher Çetinkaya Altuntaş

**Affiliations:** 1 Division of Endocrinology and Metabolism, Department of Internal Medicine Bursa Yuksek Ihtisas Education and Training Hospital, University of Health Sciences, Bursa, Turkey

**Keywords:** Anti-thyroglobulin, insulin resistance, hashimoto's thyroiditis, obesity, thyroid antibodies, thyroid peroxidase antibody

## Abstract

**Background:**

Obesity, a rapidly escalating global health concern, is associated with comorbidities and chronic inflammation. However, the link between obesity and thyroid autoimmunity remains unclear.

**Objective:**

This case-control study, conducted at a tertiary care center, aimed to elucidate the relationship between obesity and the degree of obesity, thyroid autoimmunity, and TFTs in euthyroid individuals with a BMI >30 kg/m2 and explore variations based on the degree of obesity.

**Methods:**

Free thyroid hormones, TSH, thyroid peroxidase antibodies (anti-TPO), anti-thyroglobulin antibodies (Anti-Tg), and metabolic parameters (glucose, lipid profile, insulin resistance, hemoglobin A1c) were measured in 164 euthyroid patients with obesity and 73 lean subjects aged 18-65 years. Subjects with obesity were stratified into three groups based on body mass index (BMI): first-degree obesity (BMI 30-34.9 kg/m^2^), second-degree obesity (BMI 35-39.9 kg/m^2^), and third-degree obesity (BMI ≥ 40 kg/m^2^).

**Results:**

The prevalence of thyroid antibody positivity was significantly higher in the obese group compared with the non-obese group, specifically for anti-TPO (45 (27.4%) *vs*. 7 (9.6%) and anti-Tg (35 (21.3%) *vs*. 5 (6.8%). Anti-Tg titers were elevated in the obese group (*p*=0.006), but anti-TPO levels were similar across the groups. Among the BMI-stratified groups, individuals with first and second-degree obesity exhibited higher anti-TPO positivity and anti-Tg titers compared with the control group. No significant differences were found in the third-degree obesity group. TSH and fT4 levels were higher in the obese group compared with the non-obese group (*p*=0.016 and *p*=0.045, respectively), whereas fT3 levels and the fT3/fT4 ratio remained consistent across the groups. Although no direct correlation was found between thyroid autoantibodies and metabolic parameters, individuals positive for anti-TPO and/or anti-Tg exhibited worse metabolic profiles compared with individuals who were antibody-negative.

**Conclusion:**

There is an increase in thyroid autoimmunity among euthyroid individuals with obesity; however, this increase does not appear to be proportional to BMI. The effect of antibody presence on metabolic parameters in individuals with obesity is not yet fully understood.

## 1 INTRODUCTION

Obesity is a chronic disease with a rapidly increasing prevalence worldwide, caused by an imbalance between caloric intake and energy expenditure, often influenced by genetic factors. It is associated with metabolic abnormalities, such as insulin resistance (IR) and type 2 diabetes mellitus (T2DM), as well as with certain cancers and immune-mediated diseases, including autoimmune disorders, such as multiple sclerosis (MS) and rheumatoid arthritis (RA) [1].

Hashimoto's thyroiditis (HT) is a chronic inflammatory condition characterized by T-cell infiltration of the thyroid parenchyma, followed by destruction and fibrosis, resulting in varying degrees of thyroid dysfunction. The pathophysiology of HT is not fully elucidated; however, it involves a robust immune response mediated by cytokines released by sensitized T cells and antibodies targeting thyroid antigens [2]. Recent years have witnessed a rise in the incidence of autoimmune diseases, particularly in developed and industrialized nations. Although the correlation between thyroid hormone alterations and obesity is well-established, the relationship between thyroid autoimmunity and obesity remains unclear.

The influence of overt thyroid dysfunction on body weight is well recognized, yet the impact of mild variations in thyroid function tests (TFTs) on weight changes is uncertain. Recent studies have concentrated on the association between slight modifications in serum thyroid-stimulating hormone (TSH) and free thyroid hormones with body mass index (BMI) in euthyroid individuals, though conclusive results have yet to be achieved [3, 4].

Existing literature includes a limited number of studies investigating TFTs and autoimmunity within normal reference ranges related to obesity and its components, resulting in conflicting findings. Some studies assessed only TSH, others assessed both TSH and free thyroxine (sT4) [5, 6], and some solely assessed thyroid autoantibodies. These studies often exhibit methodologic limitations, and many have been conducted on pediatric, adolescent [7-9], or pregnant populations [10], complicating the extrapolation of findings to the general population. Furthermore, studies involving adult populations have produced inconsistent outcomes [11, 12].

This research will contribute to the literature because it is the first study to explain the relationship between obesity and the degree of obesity, thyroid autoimmunity, and TFTs. This study aimed to elucidate this relationship in euthyroid individuals with a BMI >30 kg/m^2^ and explore variations based on the degree of obesity.

## 2 MATERIALS AND METHODS STUDY SAMPLE

This single-center, case-control, cross-sectional study was conducted at the University of Health Sciences Bursa Yuksek Ihtisas Training and Research Hospital's Endocrinology and Metabolism Clinic from May 2022 to May 2024. The study included 164 patients aged 18-65 years with a BMI >30 kg/m^2^ who were consecutively recruited according to the criteria defined by the World Health Organization (WHO) [13]. Patients were categorized into three groups based on their BMI: first-degree obesity (BMI 30-34.9 kg/m^2^), second-degree obesity (BMI 35-39.9 kg/m^2^), and third-degree obesity (BMI ≥40 kg/m^2^). The healthy control group consisted of 73 age- and sex-matched volunteers with a BMI between 18.5 and 29.9 kg/m^2^, who were evaluated at the endocrinology clinic and found to be free of any underlying disease. Exclusion criteria included (1): abnormal TSH values (2), pregnancy or lactation (3), acute or chronic liver or kidney disease (4), history of malignancy (5), uncontrolled and/or complicated T2DM (HbA1c >7.5% or receiving insulin therapy) (6), diagnosis of T1DM (7), uncontrolled hypertension (using more than one antihypertensive agent or having blood pressure >140/90 mm Hg) (8), BMI >50 kg/m^2^ (morbid obesity) (9), history of thyroid surgery, and (10) use of medications that could affect thyroid function tests, such as corticosteroids, amiodarone, or immunotherapy.

### 2.1 Anthropometric, Biochemical, and Hormonal Measurements

**Fig. (1):**
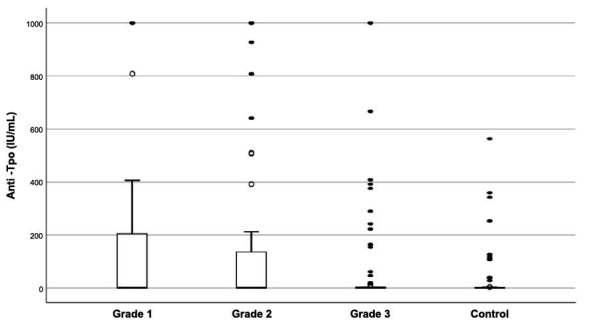
Anti-tg levels according to obesity grade. First-degree obesity (BMI 30-34.9 kg/m^2^), second-degree obesity (BMI 35-39.9 kg/m^2^), and third-degree obesity (BMI ≥40 kg/m^2^).

**Fig. (2):**
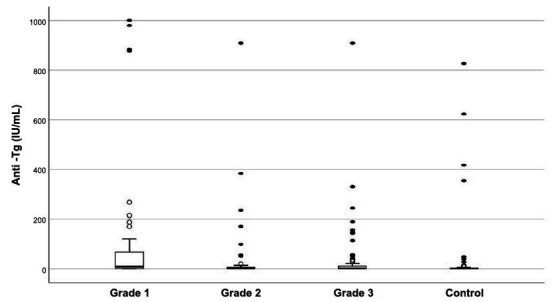
Anti-TPO levels according to obesity grade. First-degree obesity (BMI 30-34.9 kg/m^2^), second-degree obesity (BMI 35-39.9 kg/m^2^), and third-degree obesity (BMI ≥40 kg/m^2^).

Sociodemographic characteristics, including age, sex, marital status, and alcohol and smoking habits, were recorded. All participants underwent a physical examination. Body weight (kg) and height (cm) were measured, and BMI was calculated (kg/m^2^). Blood samples were collected from all participants between 08:00 and 09:00 AM after an 8-hour fasting period. The tests included TSH, fT4, free triiodothyronine (fT3), thyroid peroxidase antibodies (Anti-TPO), thyroglobulin antibodies (Anti-Tg), insulin, glucose, glycated hemoglobin A1C (HbA1c), total cholesterol, low-density lipoprotein (LDL), high-density lipoprotein (HDL), triglycerides, and Homeostatic Model Assessment of Insulin Resistance (HOMA-IR), which was calculated using the formula: fasting plasma glucose (mg/dL) x fasting plasma insulin (μU/L) / 405 [14]. The reference ranges for thyroid hormones and autoantibodies were: TSH 0.35-4.94 μIU/mL, fT3 1.58-3.91 pg/mL, fT4 0.7-1.48 ng/dL, anti-TPO 0-5.61 IU/mL, and anti-Tg 0-4.11 IU/mL. Anti-TPO ≥45 IU/mL and anti-Tg ≥45 IU/mL were considered indicative of biochemical HT because antibody positivity can be observed in the normal population. A single positive antibody was sufficient for HT diagnosis. In our laboratory, values higher than 1000 for anti-TPO and anti-Tg are reported as >1000, so the maximum value was recorded as 1000. All data were compared between the patient and healthy control groups (Figs. **1** and **2**).

Ethical committee approval was obtained from the Ethics Committee of the University of Health Sciences Bursa Yuksek Ihtisas Training and Research Hospital (Approval No: 2011-KAEK-25 2022/05-05, Approval Date: 18/05/2022). All participants were informed about the study. All procedures performed in this study were conducted according to the ethical standards of the institutional and/or national research committee and the 1964 Helsinki Declaration and its later amendments or comparable ethical standards.

### 2.2 Statistical Analysis

The Statistical Package for the Social Sciences (SPSS) version 22.0 software package was used for statistical data analysis. Categorical variables are presented as frequencies (n) and percentages (%), and continuous variables are expressed as means with standard deviations (mean ± SD, range). Categorical data were compared using the Chi-square and Fisher's exact tests, and the normality of numerical data distribution was assessed using the Kolmogorov-Smirnov and Shapiro-Wilk tests.

Student’s t-test was used for comparisons between two independent groups with normally distributed numerical variables, and the Mann-Whitney U test was used for non-normally distributed data. Oneway analysis of variance (ANO-VA) was used when comparing more than two independent groups with normally distributed numerical variables, and the Kruskal-Wallis test was used for non-normally distributed data. Post-hoc analyses for variables showing significance in the Kruskal-Wallis test were conducted using the Mann-Whitney U test with Dunn-Bonferroni correction. The relationship between non-normally distributed numerical variables was assessed using Spearman's correlation analysis. The threshold for statistical significance was set at *p*<0.05. The power of the study was calculated as 93.28% based on the analysis of data from the two independent groups.

## 3 RESULTS

The study included a total of 237 participants, comprising 164 individuals with obesity (BMI 38.74 ± 5.58 kg/m^2^, age 41.91 ± 11.31 years) and 73 non-obese individuals (BMI 24.34 ± 3.04 kg/m^2^, age 39.01 ± 9.93 years). Among the participants with obesity, TSH and fT4 levels were significantly elevated compared with the non-obese group (2.23 ± 1.37 *vs*. 1.77 ± 1.05 μIU/mL, *p* = 0.016; 1.04 ± 0.21 *vs*. 1.02 ± 0.27 ng/dL, *p* = 0.045, respectively). The fT3 levels and fT3/fT4 ratios were similar between the groups. The number of individuals with positive thyroid antibodies was statistically higher in the obese group compared with the non-obese group. Specifically, for anti-TPO, there were 45 (27.4%) *vs*. 7 (9.6%), and for anti-Tg, there were 35 (21.3%) *vs*. 5 (6.8%). Among the antibody titers, only anti-Tg levels were significantly higher in the obese group, whereas there was no statistically significant difference in anti-TPO levels. Table **1** presents the demographic, hormonal, and metabolic parameters of the obese and non-obese groups.

Further stratification of patients with obesity into three groups based on obesity grade revealed that the grade 1 obesity group (n=50, BMI 30-34.9 kg/m^2^) showed significantly higher anti-Tg positivity and titers compared with the healthy control group (17 (34.7%) *vs*. 5 (6.8%), *p* = 0.001; 131.65 ± 285.09 *vs*. 33.83 ± 133.98, *p* < 0.001). Anti-TPO positivity was higher in both the grade 1 and grade 2 obese groups compared with the control group. Table **2** demonstrates the results of subgroups.

Patients with anti-Tg positivity had significantly higher levels of TSH, glucose, and HOMA-IR compared with those with normal anti-Tg levels (*p* = 0.011, *p* = 0.001, and *p*=0.014, respectively). Patients with anti-TPO positivity had significantly higher levels of TSH, total cholesterol, triglycerides, insulin, and HOMA-IR compared with those with normal anti-TPO levels (*p* < 0.001, *p* = 0.035, *p* = 0.021, *p* = 0.006, and *p* = 0.004, respectively). Patients who were positive for both anti-Tg and anti-TPO had significantly higher levels of glucose, triglycerides, insulin, and HOMA-IR compared with other patients (*p* = 0.034, *p* = 0.037, *p* = 0.009, and *p* = 0.005, respectively). No statistically significant relationship was found between anti-Tg, anti-TPO, LDL, triglycerides, HDL, total cholesterol, glucose, HbA1c, HOMA-IR, and insulin.

## 4 DISCUSSION

This is the first case-control study to compare TSH, fT4, fT3, fT3/fT4 ratio, and thyroid autoantibody levels in individuals with obesity (BMI >30 kg/m^2^) and healthy, non-obese volunteers. In this study, TSH, fT4 levels, anti-Tg, anti-TPO positivity, and anti-Tg titers were found to be higher in the obese group compared with the non-obese group. When stratified according to the grade of obesity, anti-TPO positivity and anti-Tg levels were higher only in individuals with first and second-degree obesity compared with the control group.

The relationship between obesity and various autoimmune diseases, such as RA, MS, and psoriasis, has been well established, but the relationship with thyroid autoimmunity remains unclear. The number of studies examining the relationship between obesity and HT is limited, and the results are controversial [11, 12]. Some studies found a relationship between anti-TPO and obesity [15], whereas others did not [12]. In a study by Marzullo *et al*. (n=168), autoimmune thyroid disease was found to be higher in patients with obesity. Our study eliminates the potential confounding effect of hypothyroidism because all our subjects were euthyroid. These studies compared obese and lean individuals similar to our study. Regional differences have been reported, for example, Marzullo *et al*. (n=168) in Italy [11] and Wang *et al*. (n=2808) [12] in China with a much larger population. However, the weakness of these studies is that this study included euthyroid patients as well as those with hypothyroidism and subclinical hypothyroidism [11, 12].

Our study also found an increased frequency of HT in individuals with obesity, consistent with the literature. The exact mechanism remains unknown because adipose tissue has metabolic, inflammatory, and immunologic roles in addition to its fat-storage function [16]. Visceral adipose tissue (VAT) in the abdominal cavity is metabolically active and associated with IR and metabolic syndrome [17]. There is growing evidence that VAT is associated with low-grade chronic inflammation mediated by the immune system in individuals with obesity [18]. Therefore, there is a strong relationship between obesity, adipose tissue, and autoimmunity. Adipose tissue exhibits a shift from anti-inflammatory processes (such as decreased secretion of IL-10, IL-4, and TGF-beta and increased secretion of cytokines, such as IL-1, IL-6, and TNF-alpha) to pro-inflammatory mechanisms, resulting in decreased self-tolerance. This shift can lead to the activation of immunologic and oncogenic pathways, alongside metabolic effects such as IR [19]. The increased inflammatory markers cause vasodilation and increased vascular permeability in the thyroid gland, leading to functional and morphologic changes [20]. In the literature, studies have often focused on anti-TPO more than anti-Tg, considering the presence or absence of these antibodies without examining antibody titers. In our study, to avoid false positives, we considered anti-TPO and anti-Tg levels above 45 IU/mL as positive, given the prevalence of thyroid autoantibodies in the general population at 10-12% [21]. Interestingly, we found that although both antibodies were positive in individuals with obesity, only anti-Tg levels were significantly higher. This was not observed for anti-TPO. The mechanism behind this is unclear. We believe that this is a novel and interesting finding. Until now, anti-Tg has been considered less significant compared with anti-TPO. However, this finding suggests that anti-Tg levels could be a new marker for monitoring and identifying issues related to obesity. This area warrants further research.

Based on these results, we expected to see more HT in the group with BMI >40 kg/m^2^. However, our findings showed higher autoimmunity in groups with lower BMI. This surprising result suggests that autoimmunity increases even at the onset of obesity, but further weight gain does not elevate this risk. The threshold for increased autoimmunity appears to be at BMI >30 kg/m^2^.

Although there was no statistically significant relationship between thyroid antibodies and metabolic parameters, such as glucose, lipid profile, and HOMA-IR, patients positive for anti-Tg and/or anti-TPO had a worse metabolic profile compared with patients who were antibody-negative. In particular, those positive for anti-TPO exhibited worse lipid parameters in addition to glucose and insulin resistance compared with individuals who were positive for anti-Tg. This could be due to either obesity or the thyroid antibodies themselves. More research is needed to elucidate the relationship between thyroid antibodies and metabolic parameters.

In euthyroid individuals, the relationship between changes in TSH and free thyroid hormones and BMI has gained attention in recent years, but definitive results have not been achieved. Some studies found higher TSH levels in individuals with obesity [5, 6, 22], whereas others did not [20, 23]. The results for free thyroid hormone levels are more contradictory [11]. The increase in TSH is thought to be a result of the relationship between the hypothalamus-pituitary-fat tissue axis, independent of hypothyroidism. This positive linear relationship between TSH and BMI is explained by the compensatory increase in thyroid hormone levels to boost basal energy expenditure in obesity. It has been shown that overt hypothyroidism and subclinical hypothyroidism can lead to weight gain, HT, and changes in glucose and lipid metabolism, contributing to atherosclerotic changes.

Additionally, a high normal TSH level has been suggested to reduce basal energy metabolism, creating a hypothyroid-like state [24, 25]. These results can thus be clinically associated with obesity and its associated metabolic and cardiovascular dysfunction. Clinicians should pay attention to these patients in this respect. Consistent with previous studies, we found higher TSH levels in the obese group compared with the non-obese group despite all patients being euthyroid. This may be related to the aforementioned mechanism or antibody positivity.

The strengths of this study include the fact that all participants were euthyroid, all thyroid function tests were conducted, and this is the first study to investigate the relationship between thyroid autoimmunity and obesity in adults aged 18-65 years, classified according to BMI and metabolic parameters.

A limitation of this study is that the sample size is not large enough. Hence, longitudinal studies over a longer period could be considered to further validate the reliability of the conclusions, and multi-center studies would provide more accurate results. Another limitation is that patients should undergo thyroid ultrasound to assess parenchymal echogenicity, thyroid volume, and the presence of nodules. Additionally, the menopausal status of female patients was not considered. Since there is no potential selection bias and the findings are objective, these results can be generalized to other populations. Nevertheless, these findings need to be supported by multicenter long-term studies in different countries.

## CONCLUSION

The frequency of HT is increased in individuals with BMI >30 kg/m^2^. However, this increase is not proportional to the degree of BMI. The type and level of antibodies in individuals with HT may contribute to the metabolic parameters associated with obesity. Based on these findings, patients with obesity should be monitored for autoimmune diseases in addition to metabolic and cardiovascular issues, and their treatment should include immune system-targeted dietary and pharmacologic interventions. Obesity is increasing very rapidly in the world. Thyroid diseases are also very common. The relationship between these two diseases is a matter of interest for future studies. Therefore, new multicenter long-term randomized controlled studies are significantly required.

## Figures and Tables

**Table 1:** Demographic, hormonal, and metabolic parameters in the obese and non-obese groups.

-	**Obese** **Group** **(n=164)**	**Healthy** **Control** **Group** **(n=73)**	p <0.001***	**Females (n=219)**	**Males** **(n=18)**	***p***
**Age (years)**	41.91±11.31 (18-65)	39.01±9.93 (18-61)	0.060	40.96±10.97 (18-65)	41.72±11.23 (18-59)	0.779
**Sex** **(female/male) n (%)**	155/9	64/9	0.066	-	-	-
**Weight (kg)**	99.18±16.40 (69- 156)	65.82±9.60 (50-90)	***	88.51±20.71 (50-142)	93.8±2731 (59-156)	0.620
**BMI (kg/m^2^)**	38.74±5.58 (30.02-49.95)	24.34±3.04 (18.73-29.90)	***	34.58±8.26 (18.73-49.95)	31.07±8.12 (20.66-46.06)	0.082
**TSH (μIU/mL)**	2.23±1.37 (0-5)	1.77±1.05 (0-5)	0.016	2.10±1.31 (0-5)	1.97±1.16 (0-4)	0.841
**fT4 (ng/dL)**	1.04±0.21 (1-3)	1.02±0.27 (1-3)	0.045	1.02±0.19 (1-3)	1.10±0.49 (1-3)	0.789
**fT3 (pq/mL)**	2.90±0.52 (1-4)	2.91±0.52 (1-4)	0.802	2.90±0.50 (1-4)	2.94±0.72 (1-4)	0.370
**fT3/fT4 (pq/mL)** **(ng/dL)**	2.87±0.62 (0.32-4.54)	2.97±0.63 (0.32-4.49)	0.282	2.90±0.60 (0.32-4.54)	2.95±0.82 0.32-4.00)	0.146
**Anti-Tg titer (IU/mL) (min /max)**	68.04±194.87 (0-1000)	33.83±133.98 (0-826)	***	61.86±185.28 (0-1000)	4.46±12.78 (0-55)	0.012
**Anti-Tpo titer (IU/mL) (min/max)**	119.45±259.82 (0-1000)	27.83±92.46 (0-563)	0.087	92.18±225.29 (0-10000)	79.59±239.59 (0-1000)	0.339
**Anti-Tg (IU/mL)** **Normal n (%)** **Positive n (%)**	129 (78.7) 35 (21.3)	68 (93.2) 5 (6.8)	0.006	170 (77.6) 49 (22.4)	15 (83.3) 3 (16.7)	0.770
**Anti-** **Tpo (IU/mL)** **Normal n (%)** **Positive n (%)**	119 (72.6) 45 (27.4)	66 (90.4) 7 (9.6)	0.002	180 (82.2) 39 (17.8)	17 (94.4) 1 (5.6)	0.323
**Hashimoto's thyroiditis (%)**	54 (32.9)	10 (13.7)	0.002	61 (27.9)	3 (16.7)	0.413
**Glucose (mg/dL)**	94.41±12.57 (71-129)	84.52±10.76 (26-106)	***	92.07±12.83 (26.129)	8272±9.96 (69-99)	0.002
**LDL (mg/dL)**	120.53±3279 (40-222)	108.50±28.88 (30-176)	0.004	15.91±32.30 (30-222)	127.90±27.42 (165-245)	0.130
**Total Cholesterol** **(mg/dL)**	194.35±39.09 (31-306)	183.67±35.26 (88-272)	0.011	190.08±38.85 (31-06)	202.89±27.10 (165-245)	0.113
**HDL (mg/dL)**	51.23±15.11 (2-168)	57.92±12.0 (31-102)	***	53.62±14.89 (29-168)	49.29±11.16 (35-68)	0.157
**Triglycerides** **(mg/dL)**	132.51±63.66 (10-393)	93.57±38.69 (41-256)	***	121.24±60.83 (10-393)	11.78±46.44 (47-212)	0.733
**HbA1c (%)**	5.58±0.64 (4-7)	5.17±0.42 (4-6)	***	5.45±0.62 (4-7)	5.52±0.51 (4-6)	0.537
**Insulin (µIU/mL)**	14.17±8.32 (3-46)	6.64±2.65 (1-15)	***	12.02±7.88 (3-46)	10.26±8.16 (1-32)	0.166
**HOMA-IR**	3.36±2.19 (0.62-12.71)	1.40±0.58 (0.20-3.35)	***	2.82±2.09 (0.20-12.71)	2.11±1.7 (0.34-7.03)	0.077

**Abbreviations:** **TSH:** Thyroid-Stimulating Hormone, **fT4:** Free Thyroxine, **fT3:** Free Triiodothyronine, **Anti-Tg:** Anti-Thyroglobulin, **Anti-Tpo:** Anti-Thyroid Peroxidase, **HOMA-IR:** Homeostasis Model Assessment: Insulin Resistance, **LDL:** Low-Density Lipoprotein **HDL:** High-Density Lipoprotein, **HbA1c**: Glycated hemoglobin, **BMI:** Body Mass Index. **Note:** Normally distributed variables are expressed as mean ± SD, non-normally distributed variables as median with IQR and categorical variables as numbers with percentages (%). ***: *p*<0.001.

**Table 2:** Distribution of demographics and laboratory findings according to obesity grade.

-	**Grade 1** **Obesity** **(n=50)a** **BMI 30-34.9 kg/m2**	**Grade 2** **Obesity** **(n=45)b** **BMI 35-39.9 kg/m2**	**Grade 3** **Obesity** **(n=70)c BMI >** **40 kg/m**	**Control** **Group** **(n=72)d**	***p***	**Posthoc** ^§^
**Age (years)**	43.73±11.18 (19-65)	42.58±9.61 (22-59)	40.21±12.28 (18-65)	39.01±9.93 (18-61)	0.052	-
**Sex (female/ male) n (%)**	46/3	41/4	68/2	64/9	0.185	-
**Weight (kg)**	82.73±7.31 (69-100)	96.24±9.69 (79-125)	112.59±12.59 (90-156)	65.82±9.60 (50-90)	***	a-b: *** a-c: *** a-d: *** b-c: *** b-d: *** c-d: ***
**TSH** **(μIU/mL)**	2.24±1.41 (0-5)	2.38±1.46 (0-5)	2.13±1.28 (0-5)	1.77±1.05 (0-5)	0.064	-
**fT4 (ng/dL)**	1.05±0.29	1.04±0.19	1.02±0.14	1.02±0.27	0.322	-
	(1-3)	(1-2)	(1-2)	(1-3)	-	-
**FT3** **(pg/mL)**	2.74±0.54 (1-4)	3.03±0.41 (2-4)	2.93±0.56 (1-4)	2.91±0.52 (1-4)	0.116	-
**fT3/fT4 (pq/mL)** **(ng/dL)**	2.71±0.63 (0.32-4.00)	2.98±0.54 (1.50-4.34)	2.92±0.64 (0.50-4.54)	2.97±0.63 (0.32-4.49)	0.093	-
**Anti-Tg titer (IU/mL)** **(min/max)**	131.65±285.09 (0-1000)	45.03±149.40 (0-908)	38.31±121.72 (0-908)	33.83±133. 98 (0-826)	***	a-d: *** b-d=0.006
**Anti-Tpo** **titer (IU/mL)** **(min/max)**	143.05±269.87 (0-1000)	165.81±318.15 (0-1000)	73.13±200.74 (0-1000)	27.83±92.4 6 (0-563)	0.069	-
**Anti-Tg Normal n (%)** **Positive n (%)**	32 (65.3) 17 (34.7)	38 (84.4) 7 (15.6)	59 (84.3) 11 (15.7)	68 (93.2) 5 (6.8)	0.001	a-d: ***
**Anti-Tpo Normal n (%)** **Positive n (%)**	30 (61.2) 19 (38.8)	32 (71.1) 13 (28.9)	57 (81.4) 13 (18.6)	66 (90.4) 7 (9.6)	0.001	a-d: *** b-d:0.042
**Hashimoto's Thyroiditis n (%)**	24 (49.0)	13 (28.9)	17 (24.3)	10 (13.7)	***	a-c: 0.030 a-d: ***
**Glucose** **(mg/dL)**	92.80±11.76 (75-129)	94.11±12.55 (71-127)	95.73±13.16 (73-129)	84.52±10.7 6 (26-106)	***	a-d: *** b-d: *** c-d: ***
**LDL** **(mg/dL)**	120.59±29.49 (56-180)	122.95±35.50 (40-222)	118.92±33.53 (48-218)	108.50±28. 88 (30-176)	0.033	b-d: 0.048
**Total** **Cholesterol** **(mg/dL)**	197.11±33.01 (107-260)	193.04±43.16 (31-303)	193.24±40.68 (104-306)	183.67±35. 26 (88-272)	0.062	-
**HDL** **(mg/dL)**	53.34±21.19 (30-168)	51.57±12.03 (36-86)	49.52±11.36 (29-100)	57.92±12.5 0 (31-102)	***	a-d: 0.036 b-d: 0.018 c-d: ***
**Triglycerides** **(mg/dL)**	128.24±65.56 (10-350)	128.42±69.79 (39-393)	138.13±58.49 (49-359)	93.57±38.6 9 (41-256)	***	a-d: 0.006 b-d: 0.006 c-d: ***
**HbA1c (%)**	5.44±0.70 (4-7)	5.61±0.55 (5-7)	5.67±0.65 (4-7)	5.17±0.42 (4-6)	***	a-d: 0.042 b-d: *** c-d: ***
**Insulin** **(µIU/mL)**	12.52±6.64 (5-41)	12.93±6.97 (3-36)	16.12±9.75 (3-46)	6.64±2.65 (1-15)	***	a-d: *** b-d: *** c-d: ***
**HOMA-IR**	2.94±1.82 (1.11-10.63)	3.08±2.02 (0.62-11.26)	3.83±2.46 (0.71-12.71)	1.40±0.58 (0.20-3.35)	***	a-d: *** b-d: *** c-d: ***

**Abbreviations:** **TSH:** thyroid-stimulating hormone. **fT4** free thyroxine. **fT3:** free Triiodothyronine. **Anti-Tg:** Anti-Thyroglobulin. **Anti -Tpo:** Anti-Thyroid Peroxidase. **HOMA-IR:** Homeostasis model assessment: insulin resistance. **LDL:** Low-Density Lipoprotein. **HDL**: High-Density Lipoprotein. **HbA1c:** Glycated hemoglobin. **BMI:** Body Mass İndex. **Note:** Normally distributed variables are expressed as mean ± SD, non-normally distributed variables as median with IQR and categorical variables as numbers with percentages (%). ***: *p*<0.001.

## Data Availability

The data and supportive information are available within the article.

## LIST OF ABBREVIATIONS

Anti-Tg: Anti-thyroglobulin Antibodies
IR: Insulin Resistance
T2DM: Type 2 Diabetes Mellitus
MS: Multiple Sclerosis
RA: Rheumatoid Arthritis
HT: Hashimoto's Thyroiditis
TFTs: Thyroid Function Tests
TSH: Thyroid-Stimulating Hormone
BMI: Body Mass Index
WHO: World Health Organization
fT3: Free triiodothyronine
Anti-TPO: Thyroid Peroxidase Antibodies
HbA1c: Hemoglobin A1C
LDL: Low-density Lipoprotein
HDL: High-density Lipoprotein
HOMA-IR: Homeostatic Model Assessment of Insulin Resistance
VAT: Visceral Aipose Tissue

## STANDARDS OF REPORTING

STROBE guidelines were followed.
